# Knowledge about breast cancer and hereditary breast cancer among nurses in a public hospital^1^

**DOI:** 10.1590/0104-1169.0185.2529

**Published:** 2015

**Authors:** Carmen Maria Dornelles Prolla, Patrícia Santos da Silva, Cristina Brinckmann Oliveira Netto, José Roberto Goldim, Patricia Ashton-Prolla

**Affiliations:** 2 MSc, RN, Quimioterapia Ambulatorial, Hospital de Clínicas de Porto Alegre, Porto Alegre, RS, Brazil; 3 Specialist in Oncology Nursing, RN, Laboratório de Medicina Genômica, Hospital de Clínicas de Porto Alegre, Porto Alegre, RS, Brazil; 4 PhD, Physician, Serviço de Genética Médica, Hospital de Clínicas de Porto Alegre, Porto Alegre, RS, Brazil; 5 PhD, Biologist, Serviço de Bioética, Hospital de Clínicas de Porto Alegre, Porto Alegre, RS, Brazil; 6 PhD, Full Professor, Departamento de Genética, Universidade Federal do Rio Grande do Sul, Porto Alegre, RS, Brazil

**Keywords:** Knowledge, Nurses, Male, Nurses, Breast Neoplasms

## Abstract

**OBJECTIVE:**

To assess the knowledge of nurses involved in the care of oncology patients in a public university hospital, regarding breast cancer and hereditary breast cancer, and to verify the use of such knowledge in their daily practice.

**METHODS:**

This is a descriptive cross-sectional study. Data were obtained through a structured, self-administered questionnaire. Out of 154 nurses, 137 (88.9%) agreed to participate in the study. Two questionnaires were excluded such that 135 questionnaires were analyzed.

**RESULTS:**

The global percentage of correct answers was not associated with age (p=0.173) or degree/specialization (p=0.815). Questions were classified into categories. In categories involving knowledge of established breast cancer risk factors and indicators of hereditary breast cancer, the rate of correct answers was 65.8% and 66.4%, respectively. On the practice of genetic counseling, 40.7% of those interviewed were not sure about the definition of genetic counseling and 78.5% reported never having identified or referred a patient at genetic risk for specialized risk assessment. Practice of educational actions regarding this subject was reported by 48.5% of those interviewed.

**CONCLUSION:**

This study reinforces the need to develop qualifying actions for nurses, so that strategies to control breast cancer become effective in their health care practice.

## Introduction

Cancer is the leading cause of death due to non-transmitted diseases worldwide and thus an important public health problem both in developed countries and in underdeveloped or developing countries. Breast cancer is the most frequent type of cancer in women and the second cause of death in this population group worldwide^(^ 1 ^)^. In Brazil, it is the most frequent tumor in women of the Southeastern (69/100.000), Southern (65/100.000), Midwestern (48/100.000) and Northeastern regions (32/100.000)^(^ 2 ^)^. According to the Brazilian National Cancer Institute (INCA)^(^ 2 ^)^, estimates for 2012/2013 indicate that 52.680 new cases of female breast cancer will be identified, corresponding to the occurrence of 52 cases per 100.000 women. Despite being considered a tumor with good prognosis in most instances and if diagnosed and treated in time, breast cancer is still associated with a high mortality rate in Brazil. The most probable cause for this observation is that the disease is still being diagnosed in advanced stages, and multiple barriers to diagnosis and treatment exist for most women who rely on the public health care system^(^ 2 ^-^ 3 ^)^. 

Breast cancer is a multi-factorial disease in which genetic and environmental factors contribute to its occurrence^(^ 2 ^)^. In a small percentage of cases, a germline mutation in a high-penetrance cancer-predisposition gene is present, which can be a major determinant of the occurrence of the disease^(^ 4 ^)^. Sporadic breast cancer, which is not primarily caused by an inherited high-penetrance mutation, represents more than 90% of breast cancer cases throughout the world^(^ 5 ^)^. It is estimated that, on average, women who live until the age of 85 will have a chance of 1 in 9 of developing breast cancer^(^ 6 ^)^. Established risk factors for breast cancer include reproductive factors (early menarche, nulliparity, age at first pregnancy over 30 years, use of high-dose hormonal contraceptives, late menopause and hormone replacement therapy), increasing age, high breast tissue density and family history of cancer, especially breast cancer^(^ 2 ^,^ 5 ^-^ 6 ^)^. Additional factors that modulate breast cancer risk include nutritional factors, physical activity, history and duration of breast feeding, obesity in post-menopause, smoking, alcohol consumption, exposure to ionizing radiation and socio-economic level^(^ 2 ^,^ 6 ^-^ 7 ^)^.

Hereditary breast cancer corresponds to approximately 10-15% of all malignant breast tumors. Among these are the tumors caused by highly penetrant germline mutations in the *BRCA1* and *BRCA2* genes. Women with mutations in one of these genes present a cumulative risk of between 55% and 85% of developing breast cancer until the age of 70 and a 15% to 65% risk of developing ovarian cancer, depending of the type and location of the mutation^(^ 8 ^)^. Features of the family history that suggest hereditary predisposition to breast cancer include, among others, early age at diagnosis, multiple synchronic or metachronic primary tumors, male breast cancer and association with other tumors such as ovarian and prostate cancers^(4,8).^ In Brazil, the breast cancer screening protocol recommended by the Ministry of Health includes annual clinical breast examination for asymptomatic women aged 40-50 and bi-annual mammographic screening for women aged 50-69. Recommendations for women at high risk for developing breast cancer are less clearly defined in Brazil. Clinical breast examination (CBE) and annual mammography (MMG) have been suggested from the age of 35 years, but different protocols are usually recommended according to the specific cause of risk. There is no evidence to support breast self-examination (BSE) as an isolated strategy for early detection of breast cancer^(^ 2 ^,^ 7 ^,^ 9 ^)^. 

Nurses have a central role in the multidisciplinary team involved in the care of patients with breast cancer, as well as those at increased risk for the disease. Therefore, it is essential to invest in the education and training of nurses, both in the recognition of risk factors and in criteria for referral of patients to maximize risk-reducing practices, especially in high-risk individuals^(^ 10 ^-^ 11 ^)^. Knowledge and identification of risk factors for sporadic breast cancer and focus on risk assessment for the genetic aspects of hereditary breast cancers are key challenges for health promotion and cancer prevention within nursing practice^(^ 12 ^-^ 13 ^)^. 

## Methods

This is a descriptive transversal study performed with nurses of a public university hospital in Southern Brazil (Hospital de Clínicas de Porto Alegre, HCPA) who were involved with the care of oncology patients in their practice. The study was approved by the Research Ethics Committee of the Institution (HCPA GPPG protocol number 120507). Knowledge in the areas of breast cancer and hereditary breast cancer was assessed through a questionnaire consisting of 29 questions distributed as follows: objective questions (mostly multiple choice) about demographic data and professional training (5 questions), about cancer and breast cancer (10 questions) and about cancer genetics and hereditary breast cancer (14 questions). In relation to breast cancer, the questionnaire assessed knowledge about disease epidemiology, risk factors, diagnosis, screening and treatment. Regarding hereditary breast cancer, knowledge about diagnostic and referral criteria was assessed. Recruitment and data collection occurred between March and September of 2013. The estimated minimum sample size at baseline was 103 nurses, and the total amount of nurses who were active in clinical and surgical hospitalization, radiotherapy, chemotherapy and outpatient units and who were involved with the care of adult oncology patients in the HCPA was 154 nurses during the study period. All professionally active nurses involved with care of oncologic patients in the institution were invited for this study, and 137 (88.9%) agreed to participate. After the nurses signed informed consent, researchers provided the questionnaire, which was answered individually by each participant. Data obtained were compiled, analyzed and compared with the existing knowledge about the topics^(^ 9 ^)^. An *Excel for Windows(r)* file was created and populated with data from the questionnaires. Data were analyzed using *SPSS(r)* version 18.0 software, mainly with simple descriptive statistics. For the assessment of the normality of quantitative variables (demographic data), the Kolmogorov-Smirnov test was applied. Due to the non-Gaussian presentation of the remainder of the data, these were presented as median and interquartile intervals. The chi-square test was performed to assess the association between the overall percentage of correct answers in relation to age and specialization. Spearman correlation was used to evaluate the association between the overall percentage of correct answers and the number of years after graduation. In all analyses, *P*<0.05 was considered significant. 

## Results

Of the 154 professionals who were active in the care of cancer patients during the period, 137 nurses (88.9%) participated in the research. Two (1.4%) questionnaires were excluded due to inconclusive answers; thus, 135 questionnaires were analyzed. The overall percentage of correct answers was not associated with age (p=0.173) or specialization in oncologic care (p=0.815). However, an inverse association (rs=-0.244, p=0.04) was observed between years since the end of training and number of correct answers. The overall median of correct answers for each participant was calculated by combining both questions of knowledge about breast cancer and inherited breast cancer. The lowest percentage of correct answers by a participant was 37.9% and the highest, 91.1% (average = 67.98%: SD 8.91). The results were categorized according to the main areas of knowledge considered in the study: breast cancer and hereditary breast cancer. Age was concentrated in the 40-49 age group; number of years since graduation varied from 1 to 50 years (median=15 years) and the length of time in care of oncology patients varied from <1 to 40 years (median=10 years). Table 1 describes the main results on the questions regarding "knowledge about breast cancer"; questions were combined in blocks according to different specific subjects. All blocks in this area of knowledge had average rates of correct answers above 65%. The higher rates of correct answers were observed in the blocks dealing with diagnosis/screening and treatment (70.5% and 74.5%, respectively). In Table 2, the block of questions dealing with breast cancer diagnosis and screening, two questions presented the lowest rate of correct answers of the entire study: 2.2% and 10.4%, respectively. Detailed results for the answers to the questions related to breast cancer knowledge are available upon request.

**Table 1 -:** Knowledge about breast cancer: main results. Porto Alegre, RS, Brazil, 2013

Block of questions	Average of correct answers in block
Epidemiology	69.3
Established risk factors	65.8
Diagnosis and screening	70.5
Breast cancer treatments	74.5

**Table 2 -:** Questions about breast cancer diagnosis and screening: main results. Porto Alegre, RS, Brazil, 2013

Questions with the lowest rate of correct answers	Total number of answers to question	Number of correct answers to question	% correct answers to question
What is the age range in which mammographic screening should be performed, according to the Brazilian Ministry of Health?	135	3	2.2
What is the minimum recommended periodicity for mammographic screening according to Brazilian Ministry of Health?	135	14	10.4

In relation to knowledge about hereditary breast cancer, most of the participants (54%) reported that knowledge about hereditary breast cancer had been acquired during graduate studies, and a small percentage reported exposure to the topic in extracurricular activities or during their post-graduation courses. A group of participants (13%) reported never having received information on the topic. Nevertheless, the blocks of knowledge "characteristics of hereditary breast cancer" and "indicators of higher risk of developing hereditary breast cancer" had the highest levels of right answers (74.9% and 66.4%, respectively). Two questions presented the lowest rate of correct answers in those blocks (Table 3). Detailed results for the answers to the questions related to hereditary breast cancer knowledge are available upon request.

**Table 3 -:** Questions about hereditary breast cancer: main results. Porto Alegre, RS, Brazil, 2013

Questions with the lowest rate of correct answers	Total number of answers to question	Number of correct answers to question	% correct answers to question
Is it true that most cases of breast cancer occur due to hereditary genetic alterations?	128	34	26.6
Family or personal history of breast cancer diagnosed in male individuals increases the risk of developing hereditary breast cancer?	134	35	26.1

**Figure 1 -:**
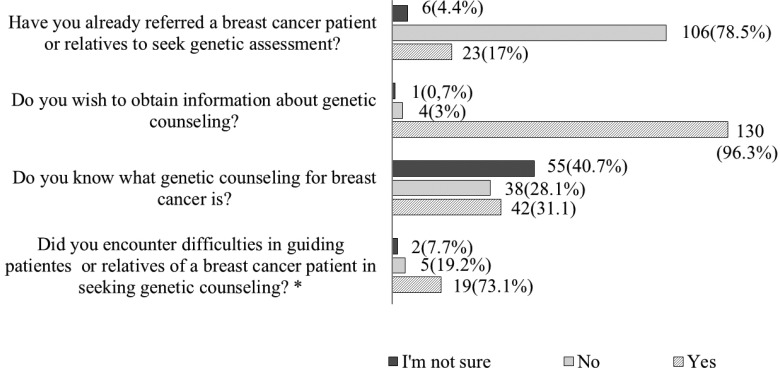
Answers to Questions about the Genetic Counseling Process for breast cancer. Total number of respondents: 135 except for * this question has N = 26 respondents.

As for the approach of including familial breast cancer history in routine anamnesis, 108 (80.6%) of nurses reported performing this approach. Still within the subject of hereditary breast cancer, Figure 1 presents the results on the questions about genetic counseling for breast cancer, where only one-third of the subjects confirmed knowing about the process. In addition, 78.5% (n=135) acknowledged never having considered referring a patient or his/her relatives to genetic risk assessment. Most of the participants, 73.1% (n=26), reported difficulties in referral to such services, including not knowing how or where at-risk patients should be referred. However, 96.3% (n=135) of the participants mentioned their interest in obtaining more information about hereditary breast cancer and genetic counseling.

**Figure 2 -:**
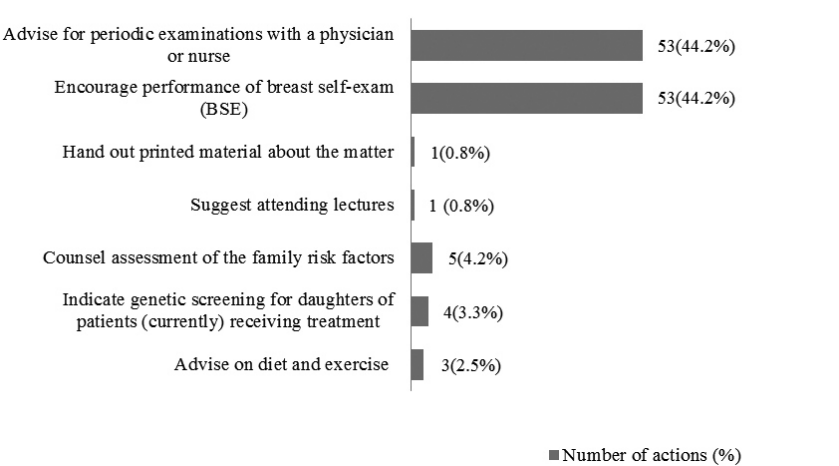
Educational and/or preventive actions effectively performed in the daily practice of the nurses. Number of actions out of a total of 120 quotes from 65 respondents.

When questioned about the professional role of nurses in carrying out educational actions to help in the prevention of breast cancer, 134 (99.3%) participants reported that these should be part of their professional activity. However, less than half (48.5%; n=65) of them effectively perform this type of preventive action in their daily professional practice. The actions effectively performed, according to reports of the participants, are described in Figure 2. 

Finally, when questioned about their interest in receiving more information about genetic risks for breast cancer and training strategies in the area, 98.5% of participants expressed their interest in training through lectures given by specialists (61.7%), seminars with discussion of illustrative cases (46.6%) and long-distance training (46.6%). 

## Discussion

The demographic data profile of participants in this study showed a predominance of professionals with considerable training and experience in the field of oncology, which is not surprising for staff nurses at a university hospital. This profile was similar to the participants in a previous study that assessed knowledge, attitudes and practice of physicians and nurses from the Family Health Strategy (FHS) program in the State of Rio Grande do Norte (city of Mossoró), Brazil, in relation to early detection of breast cancer. In that study, the average time since graduation was 17 and 15 years for physicians and nurses, respectively^(^ 14 ^)^. Although one might expect that more mature professionals would have more knowledge in this area, we observed the opposite: more experienced professionals had lower performance in the questionnaire. This finding may reflect a lack of knowledge in older professionals due to deficiencies in undergraduate training and indicates the need for the ongoing education for nurses. This need was also highlighted in the previous study^(^ 14 ^)^. 

An important fact that may explain the relative lack of knowledge of participants in specific areas is the low percentage (11%) of professionals who had taken a specialization course in oncology in this series. This fact is most likely associated with the type of activity of most nurses interviewed: generalized assistance in clinical and surgical units. In these sectors, the experience with oncology patients allows them to develop educational and assessment actions in terms of risk factors within the health/illness context, without the need for specific academic knowledge. Meanwhile, breast cancer is an action area of the nurse while also being a public health problem and an area with multiple opportunities for risk-reducing interventions^(^ 11 ^)^. Regarding the knowledge of nurses about breast cancer in general, although the overall percentage of correct answers was higher than 65%, highly compromised areas were identified, such as risk factors and current breast cancer screening strategies. 

Risk factors for breast cancer, even those established in the scientific literature, are not well known and not often discussed with women and patients in general. A study conducted in the city of Dourados (MS), Brazil, with 393 female users of the FHS Program aged 40-69 years, found that nearly half of the participants did not know any of the breast cancer risk factors and approximately 30% knew only one factor^(^ 15 ^)^. A recent case-control study about women's knowledge of breast cancer risk factors performed in a regional university in the Brazilian state of Rio Grande do Sul (RS) observed that women with breast cancer had less previous knowledge about risk factors than women without the disease and concluded that information is an important means of reducing breast cancer incidence and enabling early diagnosis^(^ 13 ^)^.

Considering that primary prevention, which aims to prevent exposure to risk factors, especially modifiable ones, such as diet and physical activity, has the potential to reduce the incidence of cancer by up to 28%, health promotion is one of the fundamental strategies to empower women to understand and intervene in determinants of their own health^(^ 16 ^)^. Within that context, the nurse has a central role, considered by some authors as a duty, in promoting the development of such educational abilities, together with the female population. Secondary prevention is also important in the control of breast cancer. An important finding of this study was the significant lack of knowledge regarding current guidelines for breast cancer screening proposed by the Ministry of Health (MH) in Brazil^(^ 2 ^,^ 9 ^)^. This observation allows us to infer that nurses might not be familiar with these protocols. The aforementioned study^(^ 14 ^)^ also identified low levels of knowledge regarding the recommended strategy of mammography screening in women. In the Mossoró study, 93.6% of the participating nurses reported that the starting age for screening mammography is 40 years. It may be that this result, as well as the low number of correct answers of the participants in the present study regarding the correct starting age and periodicity of screening as recommended by the MH, is related to the controversy over the recommendations of the MH versus those of the Brazilian Mastology Society (BMS), which recommends initiation of screening at age 40 years^(^ 17 ^)^. This recommendation is based on Law No. 11.664, of April 29, 2008, which ensures the performance of mammography in all women from the age of 40 years on, by the public health care system in Brazil, Sistema Único de Saúde (SUS)^(^ 18 ^)^, contrary to the guidelines of the MH. Finally, a revision of the barriers to access to breast cancer screening programs and the role of nursing demonstrated that the educational intervention of nurses, together with patient awareness, results in a higher patient adherence to mammographic screening^(^ 19 ^)^.

In relation to educational actions for the prevention of breast cancer described in the present study, although the vast majority of participants recognized that educational activities should be an integral component of nursing care, only half of them effectively performed these actions in their daily practice. These results are in accordance with the observations of another study on the knowledge of breast cancer in users of the public service, in the city of Bauru, São Paulo, Brazil. That work^(20) ^noted that 97.55% of the women interviewed agreed about the importance of the role of nurses as health educators, but only 35% of patients effectively received guidance from nurses regarding breast cancer prevention. 

In assessing nurses' knowledge of hereditary breast cancer and indicators of increased risk of hereditary predisposition to cancer, most of the questions had a high percentage of correct answers. The questions with a lower performance were related to frequency of hereditary breast cancer (often considered more common than it really is) and the occurrence of breast cancer in men (a frequent myth being that it does not occur)^(^ 4 ^,^ 8 ^)^. A study about the characteristics of women diagnosed with breast cancer attended in reference health services in north Minas Gerais, Brazil, indicated that 20.1% had a family history of breast cancer^(^ 21 ^)^. Another study performed in an outpatient cancer-risk evaluation program located in a teaching hospital in the state of São Paulo showed that 35.3% of women with breast cancer also had a positive family history of the disease^(^ 22 ^)^. A study performed in Porto Alegre also found a relationship between breast cancer and family history^(^ 23 ^)^. According to the study, a family history suggestive of hereditary breast cancer was identified in 6.2% of the cancer-unaffected women visiting basic health care units in the periphery of the city of Porto Alegre. 

Timely identification of patients at risk for developing hereditary breast cancer allows implementation of multiple strategies aimed at prevention or early diagnosis, both in a proband and in his/her family members^(^ 24 ^)^. The nurse involved in the care of oncology patients can be the initial identifying agent of a high-risk patient, facilitating the referral to a specialist^(^ 11 ^)^. Therefore, proper training of nurses in genetic risk identification and in the importance of referrals to high-risk programs, are crucial to enable timely referrals and use of proper risk reducing interventions^(^ 4 ^)^. Uncertainty and lack of knowledge of nurses about the role of genetic counselling and the criteria and methods for referral of patients at risk, which were all identified in this study, are an important barrier to the effective performance of these professionals.

Finally, an interesting and very positive finding of this study was the great interest nurses demonstrated in learning more about this area. A review of publications in the current bibliographic data bases (COCHRANE, LILACS, MEDLINE) in the fields of nursing and knowledge about breast cancer risk factors and screening strategies showed that they are under-represented and that the main focus is on knowledge of the patients affected by the disease. The same was evident in review articles that highlight the lack of publications in Latin America on this subject and the need for training of nurses in relation to risk factors and implementation of screening actions in routine nursing care^(^ 10 ^,^ 19 ^,^ 25 ^)^.

## Conclusion

Cancer prevention and control are among the most important scientific and public health challenges of the present. For strategies of prevention and early detection of breast cancer to result in real benefits, it is imperative to take a multidisciplinary approach, where nurses need to be aware of and knowledgeable about their educational and clinical role in the prevention and early detection of breast cancer, especially hereditary breast cancer. The assessment of knowledge and actions currently performed by nurses in this area is critical to defining the necessary training that these professionals need.
